# Hit-and-run hypothesis: revisiting the impact of EBV in malignancies

**DOI:** 10.3389/fimmu.2026.1742214

**Published:** 2026-03-24

**Authors:** Tamara Mangiaterra, Paola Chabay, Cristiana Bellan, Stefano Lazzi, Noel Onyango, Pankaj Trivedi, Paul Murray, Lucia Mundo, Eleni Anastasiadou

**Affiliations:** 1 Pathology Division, Multidisciplinary Institute for Investigation in Pediatric Pathologies (IMIPP-CONICET-GCBA), Ricardo Gutiérrez Children’s Hospital, Buenos Aires, Argentina; 2 Department of Medical Biotechnology, Section of Pathology, University of Siena, Siena, Italy; 3 University of Nairobi, Nairobi, Kenya; 4 Department of Experimental Medicine, La Sapienza University, Rome, Italy; 5 Limerick Digital Cancer Research Centre, Health Research Institute, Bernal Institute and School of Medicine, University of Limerick, Limerick, Ireland; 6 Royal College of Surgeons Ireland (Bahrain), Busaiteen, Bahrain; 7 Department of Clinical and Molecular Medicine, La Sapienza University, Rome, Italy

**Keywords:** carcinoma, Epstein-Barr virus, hit-and-run, lymphoma, viral traces

## Abstract

Epstein-Barr virus (EBV) infects more than 90% of the global population and is etiologically linked to a wide spectrum of lymphoid and epithelial malignancies. Although its role as an oncogenic virus is well established, the mechanisms underlying EBV contribution to tumorigenesis remain undefined. This review revises the “hit-and-run” hypothesis in EBV-associated cancers, proposing that the virus may initiate oncogenic transformation before leaving tumour cells. The review summarizes current evidence of EBV episomal loss, integration into host chromosomes, and the challenges associated with detecting traces of viral genetic material. Recent advances in highly sensitive detection methods, such as quantitative polymerase chain reaction (PCR), RNAscope, and single-cell droplet digital PCR, have revealed viral traces in lymphomas and carcinomas previously considered as EBV-negative by conventional *in situ* hybridization, supporting a broader role for EBV involvement in oncogenesis. Moreover, tumours with EBV traces have similar epigenetic and mutational landscapes to EBV-positive patients, suggesting that EBV-induced alterations may continue to have an impact even after EBV loss. Despite these findings, it still remains unclear whether residual viral elements contribute to ongoing oncogenic signalling, epigenetic alterations, or immune modulation within the tumour microenvironment. Investigating these factors could improve our ability to stratify patients based on EBV status, refine diagnostic criteria, and develop more targeted treatment approaches.

## 1 Introduction

In 1964, a team led by Anthony Epstein, along with Yvonne Barr and Burt Achong, discovered herpesvirus-like particles in a tumour cell line (EB1) derived from the tissues of an African Burkitt lymphoma patient (1). Later, the virus was designated Epstein-Barr virus (EBV) (2). Today, EBV, is formally classified as human herpesvirus 4 (HHV-4), a member of the gamma herpes virus subfamily, and designated as a Group 1 carcinogen by the International Agency for Research on Cancer (IARC) and the World Health Organization in the late 1990s (3).

EBV has an extraordinary host range and persists in over 90-95% of the adult human population worldwide, typically establishing lifelong latent infection following asymptomatic primary exposure during childhood. While in most individuals EBV remains clinically silent, it has been etiologically linked to a wide array of lymphoid and epithelial malignancies. The spectrum of EBV-associated cancers includes B-cell lymphomas such as endemic Burkitt lymphomas (BL), classical Hodgkin lymphoma (cHL), diffuse large B-cell lymphomas (DLBCL), post-transplant lymphoproliferative disorders (PTLD), and natural killer/T-cell lymphomas, as well as epithelial tumours such as nasopharyngeal carcinoma (NPC) and EBV-associated gastric carcinoma (EBVaGC) (4–11). Beyond oncology, EBV is increasingly implicated in the pathogenesis of autoimmune diseases such as multiple sclerosis and, more recently, primary sclerosing cholangitis, primarily through its effects on immune modulation and B cell dysfunction. Despite the substantial global burden of EBV-associated malignancies, accounting for approximately 200,000 new cancer cases annually (12), the precise mechanisms by which EBV contributes to tumorigenesis remain incompletely understood. In this review, we revisit the concept of EBV-mediated hit-and-run oncogenesis, exploring supportive evidence, and the limitations of current detection approaches.

## 2 EBV biology and latency

**Figure 1:**
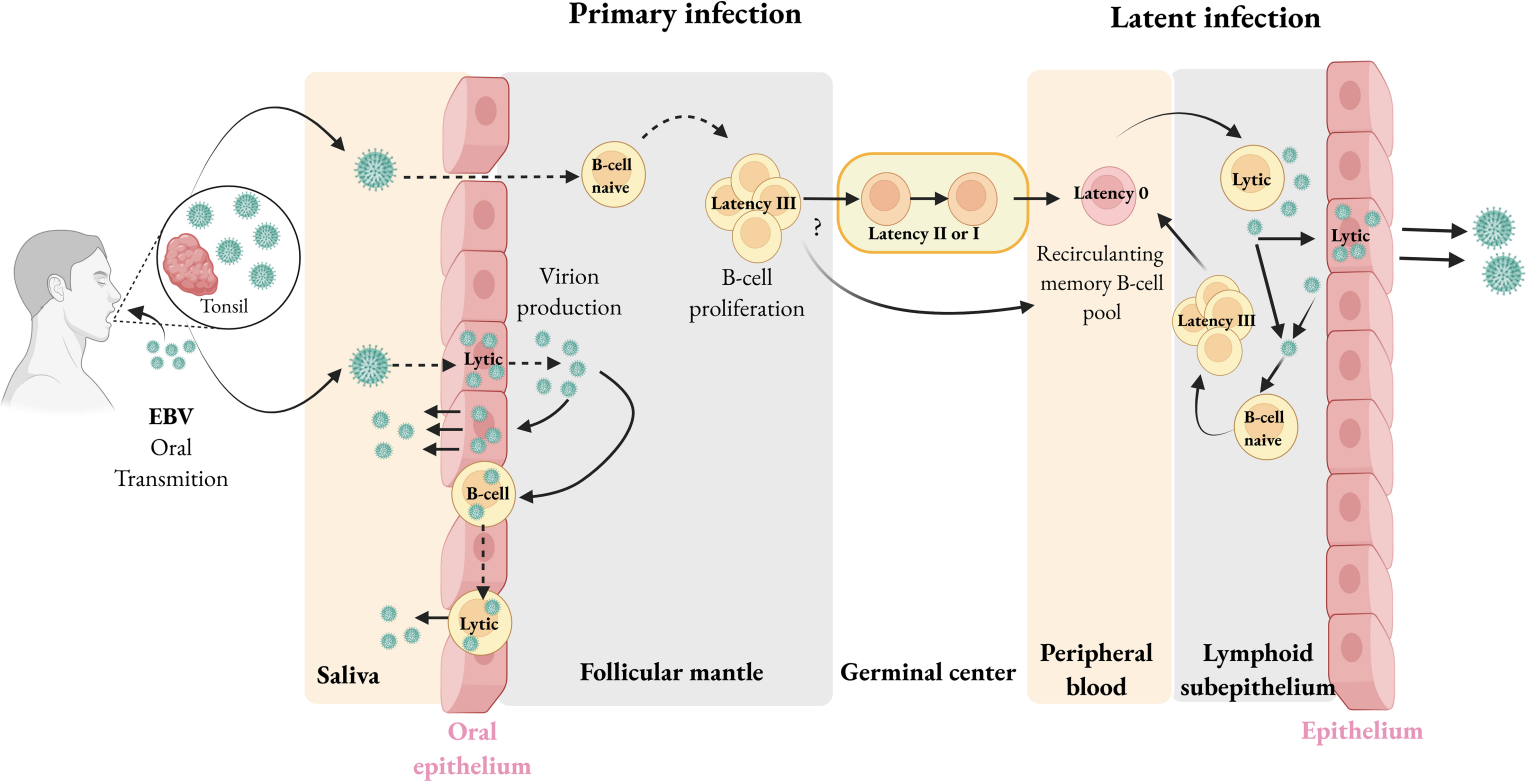
EBV infection. In immunocompetent individuals, EBV is transmitted orally and either directly infects B cells or initially infects the oropharyngeal epithelium, where it undergoes a lytic cycle before infecting B cells in the underlying crypts. During the initial phase, EBV can establish a lytic infection in B cells of the oropharynx, leading to high viral loads in saliva. Although it remains unclear which specific B cell type is initially infected, it has been proposed that EBV infects naïve B cells, expresses a latency III program, but later progressively downregulates latency gene expression to latency II and I programs to evade immune surveillance, establishing true antigenic latency (Latency 0) in memory B cells. These latently infected memory B cells circulate mainly between the blood, lymphoid tissues, and oropharynx. Occasionally, signals such as plasma cell differentiation or local mucosal cues trigger reactivation into the lytic cycle, producing new virions capable of infecting adjacent epithelial cells or B cells. Created with BioRender.com.

Most primary infections in developing countries are asymptomatic, often occurring during infancy and early childhood. In contrast, in developed countries, there’s about 50% probability of the typical symptoms known as infectious mononucleosis (IM) when primary infection occurs in young adults. Conventional non-invasive serum methods for discriminating acutely infected individuals from long-term carriers are performed using heterophile antibody, immunofluorescence immunoassay (IFI), and Enzyme-Linked Immunosorbent Assay (ELISA) tests. The virus is mainly transmitted through saliva, and primary infection is thought to occur in the oral mucosa (13–17). However, the precise origin of EBV infection during salivary transmission remains controversial. Some researchers suggest that EBV first infects oropharyngeal epithelial cells, where it replicates and releases virions, which in turn infect neighbouring B cells in Waldeyer’s ring lymphoid structures (**Figure 1**) (18). Others propose that EBV enters tonsillar crypts, crosses the thin epithelial layer, and infects naïve B cells within the follicular mantle via an as-yet-undetermined mechanism (19). Supporting this hypothesis, a prospective study detected EBV genomes in peripheral blood three weeks before the onset of symptoms, suggesting that B cells serve as the primary reservoir of EBV before its amplification in the oral cavity (20). Human primary B lymphocytes are infected through an interaction between the major viral outer envelope glycoprotein gp350/220 and the CD21 receptor on the surface of B cells, facilitated by gp42, which allows B cell entry by forming a complex with Major Histocompatibility Complex (MHC) class II molecules (21). In addition, the recently identified host receptor R9AP directly interacts with the viral gH/gL complex to initiate gH/gH/gH/gL-gB-mediated membrane fusion, acting as a critical co-receptor to promote membrane fusion and enhance EBV entry into B cells (22).

After primary infection, EBV establishes a latent infection in the peripheral blood, characterized by reduced viral production, and in this way ensures persistence with minimal impact. There are four patterns of EBV latency, which are defined based on the pattern of EBV proteins and small RNAs expressed *in vitro* infections and EBV-associated tumours (**Table 1**). The EBV genome within LCLs usually expresses all latent genes. This is known as latency III (also called the growth program), which includes six Epstein–Barr nuclear antigens (EBNA1, EBNA2, EBNA3A, EBNA3B, EBNA3C, and EBNA-LP), three latent membrane proteins (LMP1, LMP2A, and LMP2B), two small non-polyadenylated RNAs (EBER-1 and EBER-2), and transcripts from the BamHI-A region (BARTs) (23). The latency III program also occurs in iatrogenic immunodeficiency lymphoma (e.g., post-transplant lymphoproliferative disease (PTLD), primary CNS lymphoma (HIV-associated), NHLs with primary immune disorders, some cases of EBV-positive DLBCL NOS (Not Otherwise Specified), as well as DLBCL associated with chronic inflammation (20, 23).

**Table 1:** EBV latency profiles.

Latency	Antigens	EBV-associated diseases	Percentage of EBV incidence
0	EBERs	None	
I	EBERs miR-BARTs EBNA1	BL	95% (endemic)
			15-20% (sporadic)
		PEL (HIV-associated)	80%
		Plasmablastic lymphoma (HIV-associated)	60%
II	EBERs miR-BARTs EBNA1 LMP1 LMP2A LMP2B	cHL	50% - 100%
		EBV+ DLBCL	70-80%
		Lymphomatoid granulomatosis	100%
		Angioimmunoblastic T-cell lymphoma	85% - 95% large B-immunoblasts
		NK/T-cell lymphoma (nasal type)	100%
		Aggressive NK-cell leukemia	90%
		NPC	100%
		GC	10%
III	EBERs miR-BARTs miR-BHRF1 EBNA1 EBNA2 EBNA3A EBNA3B EBNA3C EBNA-LP LMP1 LMP2A LMP2B	PTLD	34% - 100%
		Primary CNS lymphoma (HIV-associated)	100%
		NK/T- cell lymphoma	100%
		DLCBL asssociated with chronic inflammation	80%
		EBV+ DLBCL	20-30%

BL, Burkitt lymphoma; PEL, primary effusion lymphoma; HIV, human immunodeficiency virus; NPC, nasopharyngeal carcinoma; cHL, classical Hodgkin lymphoma; DLBCL, diffuse large B-cell lymphoma; GC, gastric cancer; PTLD, post-transplant lymphoproliferative disorder; and CNS, central nervous system. This table outlines the different EBV latency profiles, the specific viral antigens expressed in each, the diseases commonly associated with each latency type, and the estimated percentage of EBV positivity seen in those diseases.

Latency II (also called the default program), where EBNA1 and LMP proteins are expressed, is usually seen in classical Hodgkin lymphoma, some cases of EBV-positive DLBCL, lymphomatoid granulomatosis, angioimmunoblastic T-cell lymphoma, extranodal NK/T-cell lymphoma, nasal type, aggressive NK-cell leukaemia, NPC, and gastric carcinoma (GC) (20, 23). In Latency I, only EBNA1 is expressed and is found in BL, primary effusion lymphoma (PEL), and plasmablastic lymphoma, oral type, in HIV-infected immunodeficient patients. Latency 0, where none of the EBV antigens are expressed, enables the cell to evade immune detection and is typically observed in circulating memory B cells of healthy individuals (20, 23). The expression of EBERs and viral miRNAs occurs consistently across all EBV latency types (20, 23). EBV can also reawaken periodically in response to certain triggers, shifting from latency to the lytic cycle. During this process, infectious virus particles are produced and transmitted to new hosts (23) (**Figure 1**). Some individuals may develop a chronic active EBV infection, a T-cell disorder marked by ongoing or recurrent viral replication, significantly increased levels of EBV DNA in the blood, and clinical symptoms such as infiltration of organs by EBV-infected lymphocytes (24).

## 3 EBV infection and maintenance in human cells

**Figure 2:**
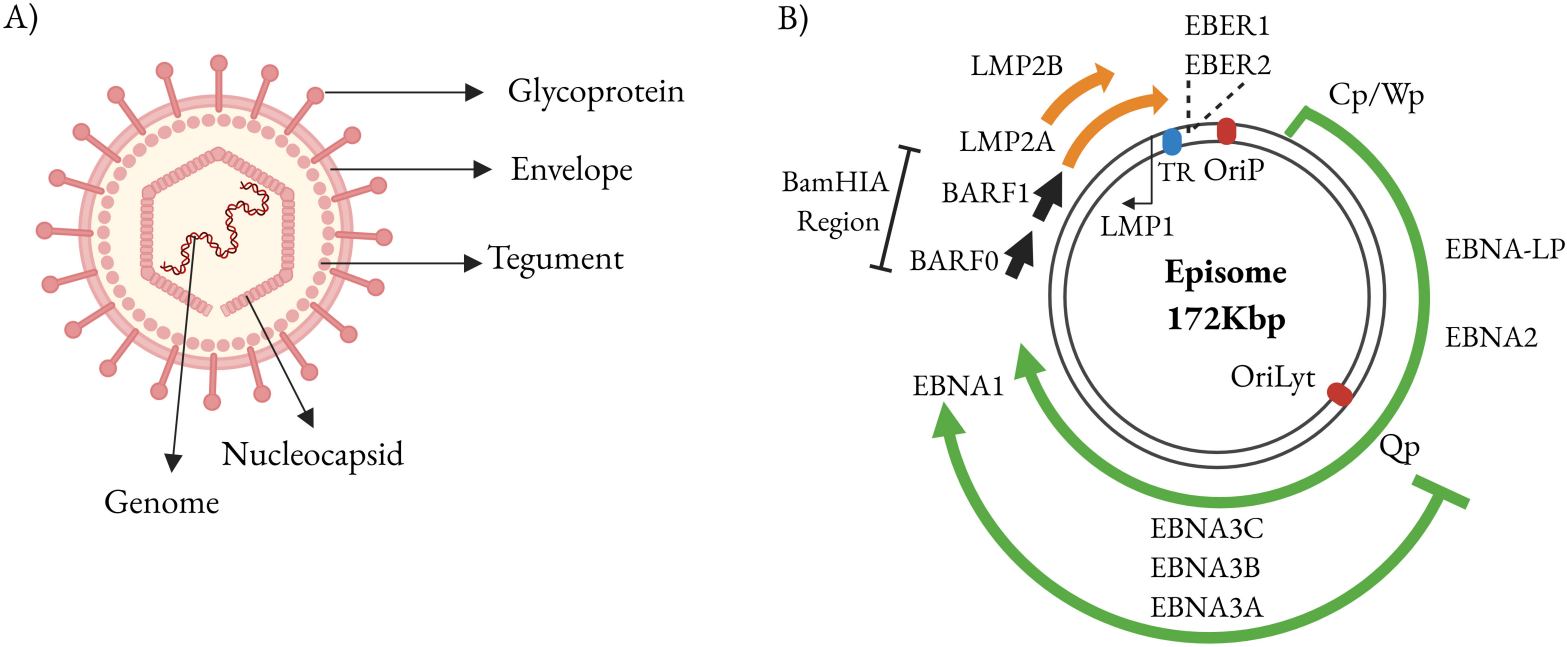
EBV genome. **(A)** Structure of the EBV genome and location of open reading frames (ORFs) encoding latency-associated proteins in the linear genome. **(B)** Diagram showing the localization and transcription of EBV latency genes in the circular episomal form of the viral genome. Created with BioRender.com.

Latency ensures that the EBV genome, a double-stranded closed-circular DNA molecule of ~170,000 bp, is maintained in the host cell as an extrachromosomal circular episome (25) (**Figure 2**). EBV episomes are associated with cellular chromosomes during mitosis and are replicated by the cell DNA polymerase during S phase (26, 27). Tethering to the host chromosome is crucial for episomal maintenance of the viral genome. Two EBV components are required to stably maintain the virus in dividing human cells: the cis-acting DNA segment known as the origin of plasmid replication (oriP), and the trans-acting EBNA-1 protein (28, 29). The C-terminus of EBNA-1 mediates specific binding to oriP DNA and increases the initial accumulation of replicated DNA but cannot mediate mitotic chromosome association or episome persistence (27, 29). The N-terminus of EBNA-1 is necessary for efficient accumulation of replicated oriP DNA, and long-term episome persistence, allowing EBV episomes to be tethered to cellular chromatin (30). This implies that mutations in the EBNA-1 basic domains may have pleiotropic inhibiting effects in enhancing oriP-dependent transcription, augmenting replicated DNA, and enabling episome persistence. In line with this, a previous study showed that deletion of EBNA-1 had almost no effect on initial DNA replication but nearly eradicated episome persistence (28, 31, 32). Since episome maintenance is fundamental to the persistence and oncogenesis of EBV, this phenomenon has been extensively studied in tissue culture using recombinant plasmids as well as in tumour-derived cell lines (33).

In tissue culture, cells expressing EBNA1 will replicate recombinant bacterial plasmid containing an EBV origin of latency replication once each cell cycle. If the plasmid carries an antibiotic resistance gene, the plasmid will be stably maintained in the presence of antibiotic selection. In the absence of antibiotic selection, a percentage of cells will lose the plasmid with each generation until the cultured cells will be plasmid-free, suggesting that EBV episomes are lost when there is no selective pressure for their maintenance (29, 30, 33). Similarly, NPC cell lines derived from EBV-positive biopsies frequently lose EBV genomes during prolonged culture (34–36). Furthermore, EBV DNA is lost after about 2 years of *in vitro* culture from some of the cells in the EBV-positive Burkitt lymphoma line (34). Ambinder and colleagues reported that episomal loss occurs when EBV enters the lytic cycle, leading to the activation of lytic genes and the subsequent downregulation of EBNA1 transcription, which is essential for maintaining episomes (37, 38).

On the other hand, Dittmer and colleagues described two consecutive and mechanistically distinct phases of episomal loss in NPC: the first, rapid and extensive; the second, slow and linearly correlated with passage number in culture. Moreover, while the viral episomes are rapidly lost entirely in the first phase, since the virus cannot establish a stable latent infection in all cells, the EBV genome is lost through deletions and successive mutations in the “slow” phase (39). The regulatory factors and molecular mechanisms behind EBV loss remain unknown; however, it can be speculated that during the “rapid-loss phase,” a host factor becomes rate-limiting, and a high EBV episome load may be disadvantageous for growth in culture. Conversely, during the “slow-loss phase,” recombination and mutation events that naturally occur can produce stable episomes. This is evidenced in some BL cell lines, which lose the EBV genome after large deletions (34, 35, 39). However, the same argument likely does not apply to other BL cell lines (Raji), which maintain the virus and require some EBV transforming function for growth in culture (35, 40, 41). Indeed, if the EBV genome is retained, these cells grow more rapidly, are more resistant to apoptosis, and are more tumorigenic in nude mice, compared to isogenic clones that have lost the virus.

EBV episomes have been shown to coexist with integrated genomes in several BL-cell lines, as well as in NPC (35, 36). Furthermore, the Namalwa cell line, derived from a case of African BL, carries two integrated copies of the EBV genome but lacks EBV episomes (35). The concept that the EBV genome might integrate into human chromosomes emerged in the mid-to-late 1970s, when Lindahl and colleagues provided evidence suggesting that a small number of integrated viral genome copies could exist in BL and NPC cells. The integrated viral genome exists predominantly as linear, sub-genomic fragments, and the viral gene transcription can still occur. Indeed, the expression of latent genes (EBNA1, EBNA2, and LMP) can be documented in cell lines carrying a single EBV-integrated copy (42). Whether EBV integration follows a random or non-random distribution remains debatable due to the absence of a systematic investigation of the EBV integration landscape on a genome-wide scale. However, Lestou et al. observed that in most cell lines carrying integrated EBV genomes, EBV integration frequently occurs at specific chromosomal bands, including 1p31, 1q43, 2p22, 3q28, 4q13, 5p14, 5q12, and 11p15 (43).

In some cases, EBV integration can be found at sites where potentially relevant cellular genes reside, for example MACF1 (involved in cell motility) in Namalwa cells, BACH2 (associated with cell differentiation and tumour suppression) in Raji cells, and REL and BCL-11A (both proto-oncogenes implicated in myeloid and B-cell malignancies) in NAB-2 cells (40, 44). The number of EBV integration sites is positively correlated with the total burden of chromosome structural variations (SVs) and copy number structural variations (CNVs). Most breakpoints are located within or near regions of genomic structural variation, suggesting that EBV integration may be driven by host genome instability, which the integration process itself could further exacerbate. Consequently, these integration sites may become chromosomal regions vulnerable to breakage, like fragile sites, potentially leading to the loss of both viral and host DNA (41, 45, 46). This raises the possibility that, under certain conditions, EBV may contribute to carcinogenesis through a “hit-and-run” mechanism, whereby the virus transiently expresses its proteins, even in linear form, before being lost due to integration into fragile genomic sites within the cell.

## 4 Hit and run hypothesis: mechanisms and challenges

The concept of virus “hit-and-run” in carcinogenesis and malignancy was first mentioned by Skinner in 1976, in the context of HSV-2 (47). According to this model, the virus could induce malignant transformation in infected cells without the need to maintain its genome once the transformation is complete. It has been proposed that viruses might act as a “hit-and-run” oncogene, meaning that the virus could drive early oncogenic events but does not need to persist in the established tumour. Moreover, this concept has been further explored in the context of several oncogenic viruses, including adenoviruses, polyomaviruses, and gamma herpesviruses (37, 48–50). This hypothesis has also been proposed for EBV, suggesting that the loss of the viral genome observed in cell lines, and potentially in patients, may contribute to disease progression (37, 40, 51).

The “hit-and-run” hypothesis proposes that the oncogenic effects initiated by a virus are later sustained by stable genetic or epigenetic alterations within the host cells, rendering the virus itself no longer essential for tumour maintenance (37, 50–52). In this scenario, the EBV episome, which is inherently prone to imperfect propagation during cell division, may be gradually lost from tumour clones. This concept suggests that EBV could play an early etiological role in tumours, but is no longer detectable at later stages, either as a virus or through its gene expression, raising the question of whether viral persistence is always necessary for cancer development. However, definitive biological evidence supporting the “hit-and-run” mechanism in oncogenesis remains lacking. This challenge is further compounded by the difficulty in conclusively proving or ruling out “hit-and-run” oncogenesis using conventional EBV detection methods.

The gold standard technique for determining EBV status in cancer includes *in situ* hybridization (ISH) for EBV-encoded RNAs (EBER), which may be complemented by a less sensitive method, such as immunohistochemistry for the viral proteins LMP1 or EBNA1 (53–55). However, these methods, which target viral RNAs or proteins, have suboptimal sensitivity and may yield false negative results due to a lack of expression, inadequate antigen retrieval procedures, RNA degradation, an inadequate signal-to-noise ratio, or issues with tissue fixation or preparation. Therefore, EBV may be missed even when present (53–55). Similarly, a molecular approach based on PCR assays raises concerns about false-negative results since hybridization to EBV DNA may be hampered by partial deletion or polymorphisms in viral DNA. On the other hand, PCR may overestimate the EBV association because it cannot distinguish between the types of cells infected (i.e., malignant vs. infiltrating lymphocytes) or may yield false-positive results due to amplicon contamination (56). A more specific approach, based on single-cell droplet digital PCR (sc-ddPCR) technology combined with single-cell isolation techniques and integrated microfluidic devices, might enable the distinction of EBV-infected malignant cells within the tumour microenvironment (TME) (57, 58).

Experimental support for a “hit-and-run” mechanism is also lacking. Several historical studies attempting to directly investigate the “hit-and-run” hypothesis in primary EBV-related tumours, such as BL and cHL, have yielded conflicting results (59). This discrepancy is particularly evident in cHL, where findings in paediatric and adult cases have differed. Two independent studies on adult cHL found no evidence of viral genome fragments in LMP1-negative or EBV-negative cases. However, in paediatric cHL, EBV was detected by qPCR in a subset of EBER-negative tumours (8 out of 24; 33%) (56). Notably, two of these tumours contained defective EBV, suggesting that in some EBV-negative cases, the virus may have evaded detection due to deletions in the viral genome. Additionally, a recent study reported two cases of adult cHL in which EBV was present in the initial tumour but absent in the relapsed cHL lymph node biopsy (59). This finding further supports the possibility that, at least in a subset of cases, EBV may contribute to tumour initiation but is not required for the sustained tumorigenic phenotype.

Recent assay developments have partially addressed the challenges of EBV detection. Whole-genome and whole-transcriptome analyses of over 2,500 cancer tissues identified a greater number of virus-positive cases than previously reported (60). Notably, EBV emerged as one of the most frequently detected viruses, highlighting the limitations of currently implemented detection methods in identifying EBV vestiges. In this context, it is essential to consider that, in addition to viral DNA, EBV encodes a large repertoire of microRNAs (or miRNAs), primarily derived from the BART and BHRF1 regions. These viral miRNAs play well-established roles in oncogenic processes and immune evasion, by modulating both viral and host gene expression, and may persist or be detectable at low levels in absence of EBV genome (56). In a recent study, miRNA profiling of EBV-positive and EBV-negative BL samples revealed “traces” of EBV infection in primary BL tumours, characterised by the presence of EBV miRNAs (EBV-miR-9-5p, EBV-miR-10-3p, and EBV-miR-19-3p), despite the absence of EBER expression (61). To further investigate the “hit-and-run” hypothesis, our group has employed a methodological strategy that combines high sensitivity (qPCR for EBV nucleic acids) with high specificity (EBER staining, EBNA1 immunohistochemistry (IHC), RNAscope for EBNA1 mRNA, and detection of viral genomes, such as EBERs). This approach was applied to a larger cohort of BL and other EBV-negative B-cell lymphomas (cHL, DLBCL, and FL), as well as corresponding BL and cHL cell lines, and gastric carcinomas (**Table 2**) (51, 56, 61–63).

**Table 2:** Comparison of conventional and non-conventional methods used for the detection of the EBV genome and its traces, highlighting their main characteristics.

	Method	Target	Advantages	Disadvantages
Conventional	EBERS ISH	EBV-encoded RNAs (EBERs)	(1)Highly reliable confirmatory test for EBV “Gold standard for EBV diagnosis”. (2) Identification of the type of infected cells in tissue. (3) Preservation of morphology	(1) High cost (2) Only applicable to tissues FFPE (3) Requires special skills (4) False negative due RNA degradation and insufficient tissue fixation or preparation (5) EBER is downregulated in oral hairy leukoplakia (6) False-positive results may occur due to antibody cross- reactivity with specific antigens
	Immunohistochemistry	EBV proteins (e.g., LMP1 or EBNA1)	(1) Easy accessibility. (2) Low cost (3) Identification of infected cells (4) Preservation of morphology	(1) Low sensitivity (2) False-positive results may occur due to antibody cross- reactivity with specific antigens (3) Overstimation due to a high background staining in the detection system (4) Applicable only to tissues FFPE
	Qualitative PCR	BamHI W fragment (repetitive regions of EBV DNA)	(1) Low risk of contamination and reduced labor costs (2) Rapid (within 1 to 2 days) (3) More reliable than serological methods in terms of evaluating EBV status in immunocompromised patients (4) Sensitive and specific across a wide dynamic range	(1) Could generate false-positive results due to improper blood sample storage and false-negative results due to the presence of nucleases (2) Lack of standardization (3) Expensive (4) Overestimation, as it does not allow the type of infected cell to be identified.
	qPCR	EBNA1, EBERS, LMP1	(1) Ability to differentiate between healthy carriers and patients with EBV-related disease based on viral load (low or high) (2) Allow for quantitative EBV DNA detection to monitor disease status. (3) Rapid (within 1 to 2 days). (4) For early intervention, it is useful in screening high-risk populations and in monitoring EBV reactivation. (5) Sensitive and specific across a wide dynamic range	(1) Could generate false-positive results due to improper blood sample storage and false-negative results due to the presence of nucleases (2) Lack of standardization (3) Expensive (4) Overestimation, as it does not allow the type of infected cell to be identified.
Non-conventional	RNAscope	EBERS, miRNAs, and EBV fragments (e.g., LMP1, EBNA1, among others),	(1) High sensitivity and specificity (2) Preservation of morphology (3) Simultaneous detection of viral and cellular transcripts (4) Applicable to FFPE, fresh, blood, cell line, among others samples	(1) High cost (2) Requires good RNA quality (3) Semi-quantitative analysis
	qPCR	miRNAs (EBV-miR-9-5p, EBV-miR-10-3p, and EBV- miR-19-3p)	(1) High sensitivity and specificity (2) Quantitative analysis (3) Applicable to FFPE fresh, blood, or exosome samples. (4) Potential non-invasive biomarker	(1) Does not indicate cell location (2) Limited to known targets
	Droplets digital PCR	BamHI-W fragment and EBNA1	(1) High sensitivity and precision (2) Absolute quantification (3) Lower technical variability (4) Use for various types of samples.	(1) High cost (2) Does not identify the type of infected cell (3) Limited to known targets (4) High-quality nucleic acid extraction
	Double viewRNA ISH	LMP1 and EBNA2 RNAs	(1) High sensitivity and specificity (2) Simultaneous and specific detection of two viral transcripts in the same cell (3) Preservation of morphology	(1) Applicable only to tissues FFPE (2) High cost (3) Dependent on the tissue’s quality (4) False negatives

PCR, polymerase chain reaction; ISH, *in situ* hybridization; FFPE, formalin-fixed paraffin-embedded; qPCR, quantitative polymerase chain reaction; and IFA, immunofluorescence assay; ddPCR, droplet digital polymerase chain reaction; qPCR, quantitative polymerase chain reaction; ISH, *in situ* hybridization; and miRNAs, microRNAs.

Each conventional and non-conventional EBV detection technique has intrinsic advantages and limitations. In the context of the hit-and-run hypothesis, one of the main challenges is the risk of false-positive results, especially when applying highly sensitive methods. PCR-based approaches can detect extremely low viral copy numbers, but cannot discriminate between EBV sequences present in neoplastic cells and within the tumour microenvironment. Similarly, the detection of EBV-encoded microRNAs or residual viral DNA may reflect remnants of a previous infection; however, it remains to be determined whether these traces have an active biological contribution to tumour initiation. To minimize misinterpretation, evidence supporting a hit-and-run mechanism should ideally be based on the integration of complementary methodologies, including *in situ* techniques, spatially resolved analysis and, where possible, single cell approaches. Methylation and mutation analyses can further support the hypothesis. It is important to highlight that the presence of EBV traces should be interpreted with caution, especially when they are based on a single detection method and assess whether viral signals are consistently localized in malignant cells. In our studies, the positivity of EBV traces was first checked by conventional methods and then confirmed by highly sensitive *in situ* methods which spatially demonstrated the presence of EBV vestiges in a subset of neoplastic cells.

Using this refined detection strategy, EBV was identified more frequently than previously recognised, with approximately 80% of BL, 50% of HL, 37% of DLBCL, and 6% of FL cases exhibiting evidence of EBV presence, detected by sensitive methods, within neoplastic cells (56). These findings suggest a more extensive pathogenic role for EBV in early tumour development than previously assumed. Supporting this notion, our analysis of one DLBCL and two cHL cases demonstrated a transition from EBER-ISH positivity at disease onset to EBER-ISH negativity at relapse. Additionally, our group identified a case of DLBCL characterized by an area of strongly EBER-positive cells separated from another entirely negative. Significantly, the EBER-positive lymphomas and their corresponding EBER-negative relapses were clonally related (56). These findings align with recent studies that have detected viral traces not only in EBV-negative lymphomas by EBERs *in situ* hybridization (51), but also in epithelial malignancies such as gastric cancer, which is often classified as EBV-negative (63) (**Table 3**). Specifically, in the study by Mangiaterra and colleagues (51), which analysed DLBCL cases initially considered as EBV-negative by conventional techniques, the use of more sensitive strategies, such as qPCR for EBNA1 coding region, LMP1, qualitative PCR targeting the repetitive BamHI W fragment, and Double View RNA ISH for LMP1 and EBNA2 transcripts, allowed the detection of viral traces in approximately almost one-third of cases (**Table 3**). However, the expression profile of immune response genes in cases with EBV traces showed no significant differences between cases with and without viral remnants. Remarkably, only EBV+ DLBCL defined by conventional methods, displayed differences in immune response gene expression (62). This could suggest that the mere presence of viral remnants may not be sufficient to elicit a cytotoxic immune response, as has been reported in EBV-positive lymphoma cases (64), along with increased PD-L1 expression. However, it was demonstrated *in vitro* that EBNA2 viral protein modulates the tumour microenvironment in EBV-positive B-cell lymphomas by regulating miRNAs, including downregulating miR-34a to increase PD-L1 expression and inducing miR-24 to suppress ICOSL (CD275), collectively promoting immune evasion and tumour survival (65, 66). Alternatively, it is possible that the “hit-and-run” mechanism is not sufficient to alter the local immune environment and that a sustained presence of EBV-infected tumour cells is required to trigger such responses (64). To our knowledge, Mangiaterra et al. (62) were the first to evaluate the presence of EBV viral traces in relation to immune response markers in DLBCL. However, the role of EBV traces in the pathogenesis of DLBCL, as well as in other EBV-associated neoplasias, needs to be further elucidated.

**Table 3:** Studies analysing viral traces using non-conventional methods.

Study group	Lymphoma types	Non-conventional methods	% of EBV-trace positive cases among EBV-negative samples by conventional methods
Mundo L. et al (61),	Burkitt lymphoma	miRNAs expression profile	100%
Mundo L. et al (56),	Burkitt lymphoma	qPCR/ddPCR/RNAscope	67%
	Classic Hodgkin lymphoma	qPCR/ddPCR/RNAscope	50%
	Diffuse large B-cell lymphoma (DLBCL)	qPCR/ddPCR/RNAscope	37%
Siciliano M.C. et al (63),	Gastric adenocarcinoma (ADK)	ddPCR/RNAscope	31%/15%
	Gastric cancer with lymphoid stroma (GCLS)	ddPCR/RNAscope	86%/43%
Mangiaterra T. et al (51),	DLBCL	Double viewRNA ISH	26%

Characteristics of cancer types, non-conventional detection techniques employed, and the percentage of cases showing the expression of viral traces among those classified as negative by conventional methods.

Currently, indirect evidence of EBV loss can be obtained through methylation and mutation analyses. Since oncogenic events initiated by EBV may be heritable and may persist even after the virus has been eliminated, these studies may provide insights into prior EBV infection (24, 52, 67). However, confirming that EBV contributed to tumour development after its disappearance remains challenging. This notion is supported by our investigations into the methylation status of genes such as MGMT and CDH1, which are known to be epigenetically deregulated by EBV in BL, HL, and DLBCL. Our findings revealed that cases containing EBV traces displayed methylation profiles similar to EBV-positive tumours, particularly showing promoter hypermethylation of EBV-associated target genes such as *MGMT* and *CDH1*, affecting tumour suppressor and DNA repair related genes. On the other hand, EBV negative cases lacking viral traces exhibited low methylation patterns, as previously reported (56). Moreover, the overall burden of somatic mutations-including both nonsynonymous and silent/non-coding mutations-was significantly lower in EBER-positive cHL as well as in cases with EBV traces compared to EBER-negative cases (56). These findings align with a study by Grande and colleagues, who demonstrated that mutations in apoptotic genes, including TP53, can compensate for the loss of EBV gene expression in BL (68). Furthermore, Kondo and colleagues demonstrated that in gastric carcinoma, some genetic/epigenetic alterations were shared between EBER-positive and -negative components, suggesting that EBV was eliminated from tumour cells during progression, which might be beneficial for tumour progression and/or immune evasion (69).

## 5 Conclusion

**Figure 3:**
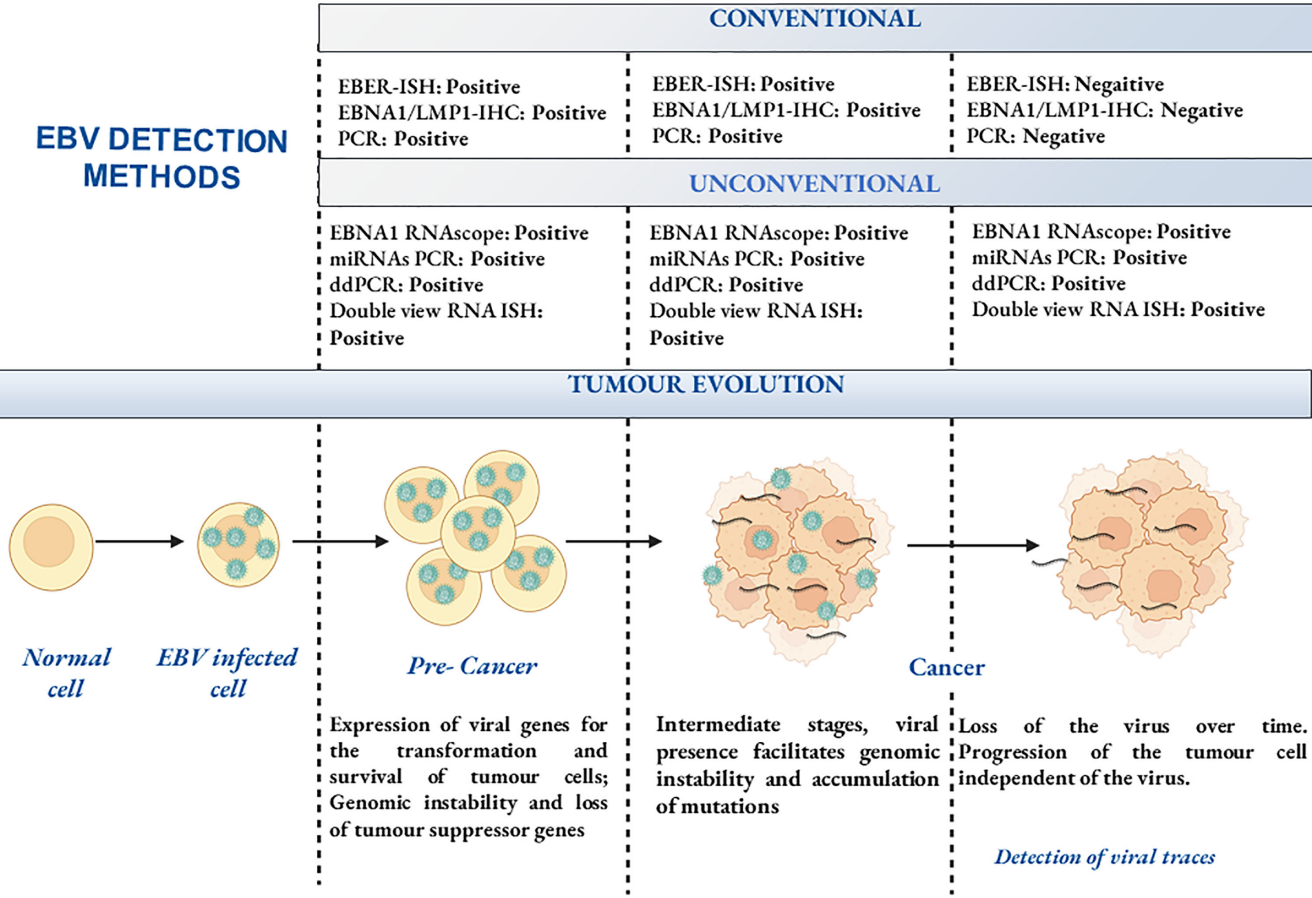
Viral hit and run oncogenesis. Schematic representation of the hypothetical hit and run mechanism and techniques for the detection of the EBV genome and its traces. Created with BioRender.com.

In summary, while EBER *in situ* hybridisation (ISH) remains the gold standard for identifying EBV-associated tumours, there is currently no definitive method to determine whether a virus-negative tumour was previously EBV-positive, or to establish the timing and extent of the virus’s involvement in the tumorigenesis (**Figure 3**). However, the more sophisticated assays discussed here have so far proved useful in retrospectively tracking the fate of the EBV genome in transformed cells and have provided further evidence of the “hit-and-run” mechanism. Future advances will likely shed further light on the mechanisms driving EBV loss in tumours. Understanding these processes could have significant implications for tumour classification, particularly in cases in which EBV status influences disease prognosis and therapy. The detection of EBV traces within tumours raises critical questions regarding its potential role in tumour biology. It still remains unclear whether residual viral elements contribute to ongoing oncogenic signalling, epigenetic alterations, or immune modulation within the tumour microenvironment. Investigating these factors could improve our ability to stratify patients based on EBV status, refine diagnostic criteria, and develop more targeted treatment approaches.

Going forward, to definitively establish or disprove a ‘hit-and-run’ mechanism in EBV-associated lymphomagenesis, approaches capable of capturing transient viral involvement and, in particular its consequences for disease pathogenesis are required. Advances in spatially resolved and single-cell technologies are expected to play a key role, as they will allow the identification of tumour subclones that may retain EBV-induced viral remnants or molecular footprints. In this context, stable epigenetic alterations, including characteristic DNA methylation patterns, as well as persistent host transcriptional changes, represent particularly promising targets, as they may reflect early EBV-driven events even after viral loss. In addition, further studies using diagnostic samples will be critical to determine whether detectable EBV loss confers a selective advantage during tumour progression.
